# Carbohydrate ingestion does not suppress increases in fatty acid-binding protein 4 concentrations post-acute aerobic exercise in healthy men: a randomized crossover study

**DOI:** 10.1186/s13102-024-00852-2

**Published:** 2024-03-04

**Authors:** Shigeharu Numao, Ryota Uchida, Takashi Kurosaki, Masaki Nakagaichi

**Affiliations:** National Institute of Fitness and Sports in Kanoya, 1 Shiromizu, 891-2393 Kanoya, Kagoshima Japan

**Keywords:** Fatty acid binding protein, Carbohydrate, Insulin, Lipolysis, Free fatty acids

## Abstract

**Background:**

Fatty acid-binding protein 4 (FABP4) has been associated with cardiovascular disease and diabetes. Acute aerobic exercise increases circulating FABP4 concentrations, but the underlying mechanisms remain unclear. The purpose of this study was to investigate the effects of inhibition of lipolysis by carbohydrate ingestion on circulating FABP4 concentrations during and after acute aerobic exercise in healthy men.

**Methods:**

Men aged between 20 and 40, with no exercise habits and no metabolic diseases, were recruited. In a randomized crossover design, the participants underwent a carbohydrate-ingestion exercise (CE) and a fasted exercise (FE) trial. The CE trial consisted of 40-min acute aerobic exercise with ingestion of carbohydrates and 60-min bed rest. The FE trial followed the same protocol as the CE trial but without carbohydrate ingestion. Venous blood samples were collected to measure hormones (adrenaline, noradrenaline, and insulin) metabolites (glycerol, free fatty acids, and glucose), and FABP4 concentrations. Ventilation and gas exchange were also collected to measure substrate oxidation.

**Results:**

Thirteen healthy men participated in and completed both the CE and FE trials. The insulin concentration was more than 4 times higher in the CE trial than in the FE trial (*p* < 0.004, effect size [ES] > 2.00). Free fatty acid concentrations were more than 4 times lower in the CE trial than in the FE trial (*p* < 0.02, ES > 2.04). However, there was no significant difference in the changes in circulating FABP4 concentrations between the CE and FE trials (*p* = 0.108), which did not change during aerobic exercise and significantly increased post-aerobic exercise in both trials (*p* < 0.002, ES > 1.212). Changes in FABP4 concentrations following aerobic exercise were not significantly correlated with changes in glycerol or free fatty acid concentrations during aerobic exercise.

**Conclusions:**

The results suggest that suppression of lipolysis and elevation of insulin are not strongly involved in increases in FABP4 secretion following acute aerobic exercise.

## Background

Fatty acid-binding protein (FABP) 4, also known as adipocyte FABP or adipose protein 2, facilitate the transport of fatty acids to specific organelles in the cell for lipid oxidation in the mitochondrion or peroxisome; lipid-mediated transcriptional regulation in the nucleus; signalling, trafficking, and membrane synthesis in the endoplasmic reticulum; and regulation of enzyme activity and storage as lipid droplets in the cytoplasm [1]. FABP4 is expressed in adipocytes and macrophages [2–4], from where it is secreted into the circulation before ultimately being eliminated by the kidneys [5, 6]. Expression of FABP4 is highest within the adipocytes [7, 8]. High circulating FABP4 levels are associated with elevated risks for various diseases [1, 9]. Short-term exogenous FABP4 administration increases hepatic glucose production and attenuates glucose disposal [7, 10]. Acute exposure to FABP4 also depresses contraction amplitude in isolated cardiomyocytes [11]. Thus, it is important to prevent over-physiological increases in circulating FABP4 concentration for various diseases prevention.

Chronic and acute aerobic exercise has beneficial effects on metabolic disorders via change in blood parameters [12, 13]. Blood parameters such as inflammatory cytokines may exhibit differential responsiveness to acute and chronic exercise. In other words, it is often observed that these parameters decrease with chronic exercise and transient increase with acute exercise [14]. It seems that this phenomenon is also observed with circulating FABP4 concentrations. While chronic exercise has been suggested to decrease circulating FABP4 concentrations [15], acute exercise has been reported to increase circulating FABP4 concentrations [16–18]. Indeed, although studies on the effects of acute exercise on circulating FABP4 concentrations are limited, the approximately 10-min acute exercise above the aerobic threshold (AT) and incremental maximal exercise increases circulating FABP4 concentration during exercise in healthy adults [16, 17]. There are also observations of increased FABP4 concentrations following exercise even in the absence of increases during exercise itself [16, 18]. The acute exercise below AT increases circulating FABP4 concentrations 10–20 min post-exercise [16]. Additionally, 40-min acute exercise at an intensity of 40% peak oxygen uptake (VO_2_peak) enhances circulating FABP4 concentrations 30–60 min post-exercise [18]. FABP4 secretion is regulated by activation of lipolytic pathway [7, 8, 19, 20]. FABP4 is released from adipocytes into the circulation through lipolysis that involves activation of the β-adrenergic receptor-mediated adenylyl cyclase–protein kinase A and natriuretic peptide receptor-A-mediated guanylyl cyclase–protein kinase pathways [7, 8, 19, 20]. Therefore, it is possible that increased circulating FABP4 concentrations during and following aerobic exercise may be due to lipolytic stimulation induced during aerobic exercise.

Inhibition of lipolysis via suppression of activation of the lipolytic pathway is induced by insulin [21]. Since carbohydrate ingestion leads to increased insulin secretion, thereby suppressing lipolysis [21], carbohydrate ingestion before and during aerobic exercise suppresses lipolysis during aerobic exercise [22, 23]. Consequently, carbohydrate ingestion would suppress circulating FABP4 levels during and following aerobic exercise. However, whether carbohydrate ingestion affects circulating FABP4 concentration during and following aerobic exercise remains unclear. Determining the factors involved in the regulation of FABP4 levels induced by aerobic exercise should contribute to elucidating the mechanism of exercise-induced FABP4 secretion. Moreover, an understanding of the underlying mechanisms may assist in the development of FABP4 as a biomarker of physiological responses.

To identify factors involved in the regulation of circulating FABP4 concentrations during and following aerobic exercise, we aimed to investigate the effects of carbohydrate ingestion on changes in circulating FABP4 concentration both during and after aerobic exercise in healthy men. We hypothesized that carbohydrate ingestion would promote insulin secretion, inhibit lipolysis, and suppress circulating FABP4 levels following aerobic exercise.

## Methods

### Study design and participants

**Fig. 1:**
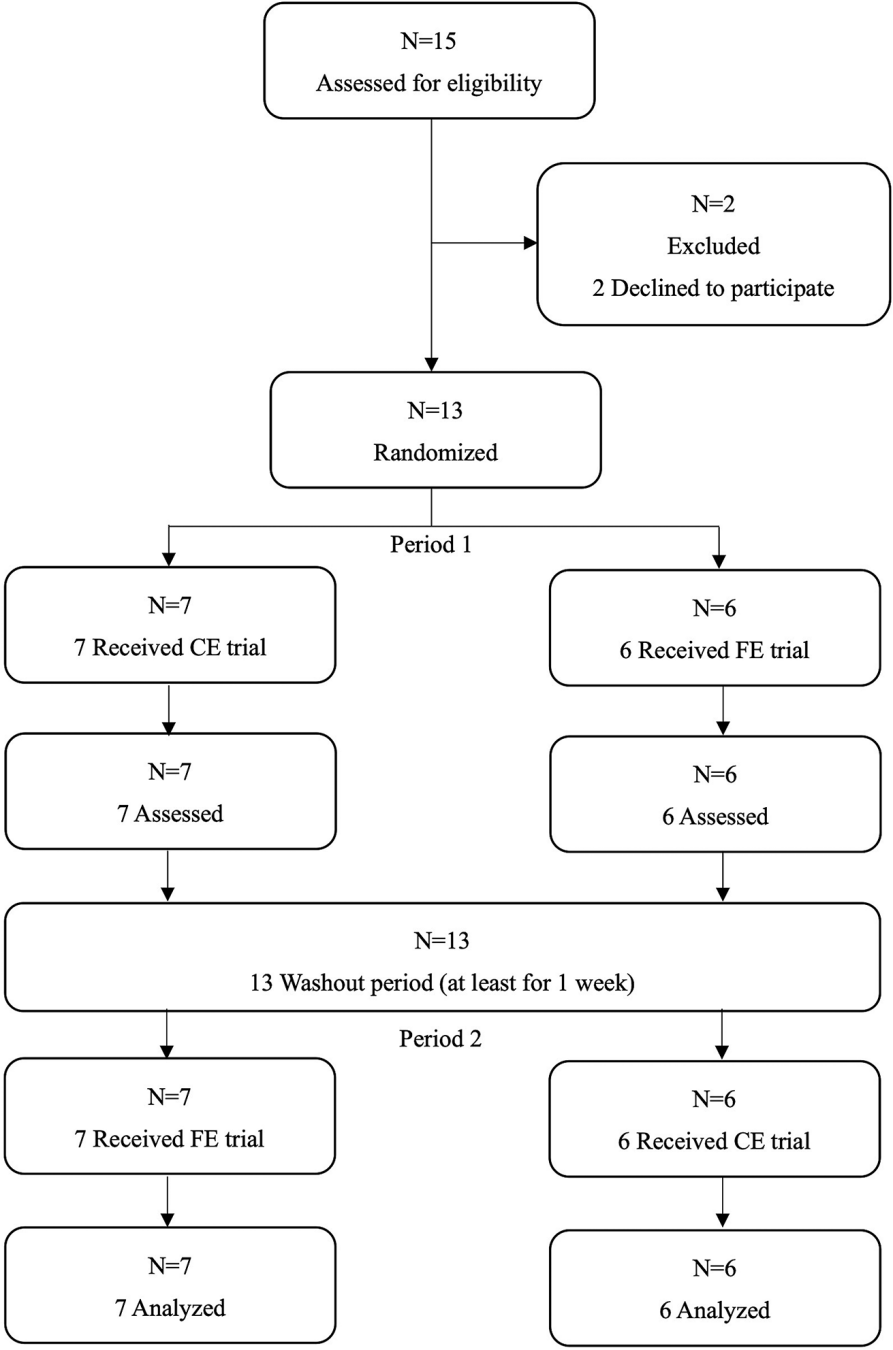
Consolidated standards of reporting trials flow diagram for crossover trials. CE,a carbohydrate ingestion exercise trial; FE, a fasted exercise trial

This study was a randomized crossover trial that adhered to CONSORT guidelines [24]. The study adhered to the guidelines outlined in the Declaration of Helsinki, and the research protocol received approval from the institutional ethics committee of the National Institute of Fitness and Sports in Kanoya (approval number 22-1-5). The study was pre-registered with the University Hospital Medical Information Network Center (UMIN), a clinical trial registration system (ID: UMIN000048052). Enrollment began in April 2022 and ended in June 2022 through campus advertisements. The experiments, including preliminary testing and the main two trials, were conducted between July 2022 and September 2022. After providing a detailed explanation of the study’s purpose, design, protocol, and potential risks, each provided written informed consent from 13 young healthy men. Exclusion criteria included: (1) women (biological sex), (2) age < 18 or > 40 years, 2) regular exercise training habits, (3) consumption of medication known to affect lipid and carbohydrate metabolism, and (4) current smoking. Given the potential influence of hormonal fluctuations and changes in body composition due to the menstrual cycle in women, this study exclusively included men to minimize potential confounding factors in the results. The participant flow diagram is depicted in Fig. 1. The lead investigator enrolled the participants in the research and randomly assigned them to each experiment (carbohydrate ingestion + aerobic exercise (a carbohydrateingestion exercise (CE) trial) or fast + aerobic exercise (a fasted exercise (FE) trial)) using computer-generated random numbers (Microsoft Excel, Microsoft, USA).

### Preliminary testing

Participants reported to our laboratory at least 1 week before the initial main experimental trial for baseline data assessment. Height was measured to the nearest 0.1 cm using a stadiometer. Weight, fat mass, fat-free mass, and skeletal muscle mass were measured to the nearest 0.1 kg using a dual-frequency body composition monitor (Inbody770; InBody Japan Inc., Tokyo, Japan). Body mass index was calculated as the weight in kilograms divided by the square of the height in meters. Blood pressure (systolic and diastolic) was measured using an automatic sphygmomanometer (HEM-1040, Omron Corp., Kyoto, Japan) after participants rested in a sitting position for 15 min. Aerobic capacity (VO_2_peak) was determined via an incremental exercise protocol with 15-watt (W) increases every 1 min after a brief warm-up period on a cycle ergometer (Aerobike 75XLIII, Konami Sports Life, Kanagawa, Japan). During the test, ventilation and gas exchange were measured using indirect calorimetry (K4b2, COSMED, Rome, Italy). The criteria for achieving VO_2_peak have been described previously [25]. The highest VO_2_ value achieved over 30 s was determined as the VO_2_peak.

### Study protocol and procedures

**Fig. 2:**
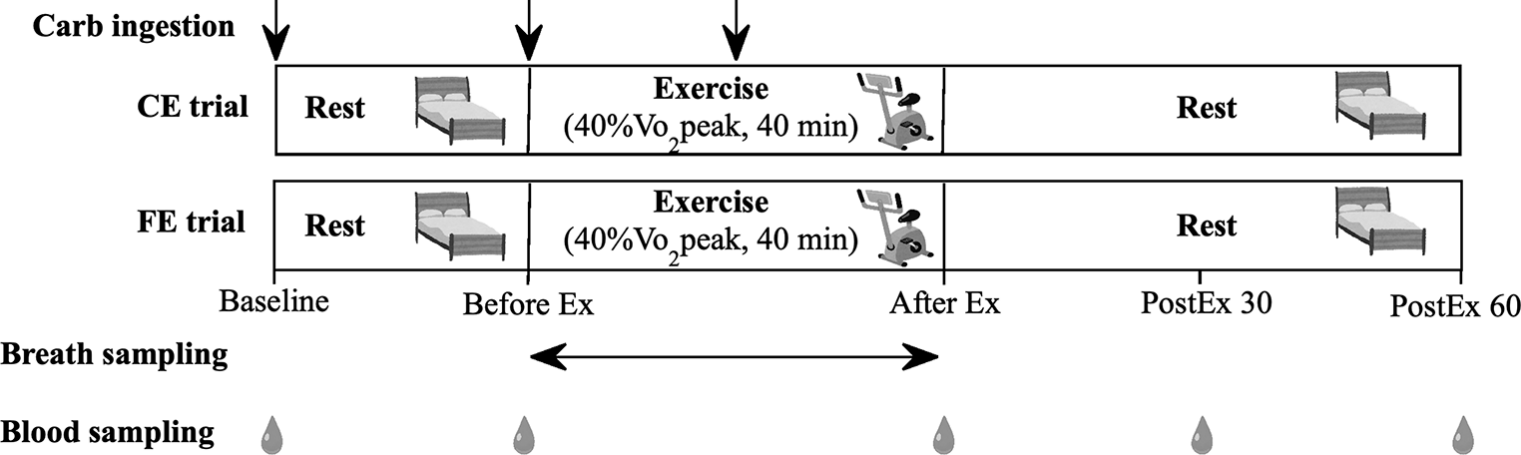
Experimental protocol

The study protocol is shown in Fig. 2. The participants reported to our laboratory three times at intervals of at least one week to eliminate any potential carry-over effects. On the first day, they underwent preliminary testing (body composition, blood pressure, and aerobic capacity). On the remaining two days, they were randomly assigned to one of two experimental trials in a counterbalanced manner. The two experimental trials consisted of (1) CE trial and (2) FE trial. After an overnight fast for at least 12 h, participants arrived at our laboratory at 7:45 AM or 9:45 AM. The arrival time was set to the same time across the two trials for each participant. After each participant rested in the supine position for 10 min, a fasting blood sample was taken. In the CE trial, participants then ingested maltodextrin jelly (0.8 g/kg body weight) within 5 min. They then rested in a supine position on the bed for a further 30 min until the start of a cycling exercise. Participants ingested a further amount of maltodextrin jelly (0.4 g/kg body weight) at the start of the cycling exercise, comprising 40 min at a workload corresponding to 40% VO_2_peak. They ingested a further amount of maltodextrin jelly (0.4 g/kg body weight) 20 min into exercise. The FE trial had the same setting as the CE trial, except that participants remained fasted throughout the trial. After both exercises, participants rested in the supine position on a bed for 60 min and drank mineral water freely. The amount of carbohydrate intake to inhibit lipolysis was determined based on previous studies [22, 23]. Furthermore, the exercise intensity and duration were set based on a previous study where circulating FABP4 concentrations exhibited the highest reactivity to exercise [18]. Baseline and during aerobic exercise in the two trials, ventilation and gas exchange were measured using indirect calorimetry (K4b2, COSMED; Rome, Italy) at -5–0, 7–10, 17–20, 27–30, and 37–40 min from the start of exercise. Based on the mean of these data, energy expenditure (EE) and carbohydrate and fat oxidation rates were calculated from VO_2_, VCO_2,_ and respiratory exchange ratio (RER) [26] every 10 min. Blood samples were collected from each participant at baseline, immediately before exercise, immediately after exercise, and 30 and 60 min post-exercise in both trials. Participants were instructed to consume the same meals for three days before each trial and to refrain from vigorous physical activity for 24 h before each trial.

### Blood sampling and analysis

Blood samples were collected in 9-mL tubes containing thrombin and 7-mL and 2-mL tubes containing sodium EDTA. The 9-ml tubes were centrifuged at 3000 *g* for 10 min at room temperature after 30 min of collection. The 7-mL tubes were centrifuged at 3000 *g* for 10 min at 4 °C immediately after collection. The corresponding serum and plasma samples were transferred into plastic tubes and immediately stored at − 80 °C until further analysis. Blood in the 2-mL tubes was used to measure hemoglobin and hematocrit values to assess changes in plasma volume [27].

Venous blood samples were collected at baseline to determine plasma epinephrine and norepinephrine and serum insulin, total cholesterol (TC), high-density lipoprotein cholesterol (HDLC), triglyceride (TG), creatinine (Cre), glucose, free fatty acid (FFA), glycerol and FABP4 concentrations. Low-density lipoprotein cholesterol concentrations were estimated following Friedewald [28]. The remaining blood samples, except for the baseline blood samples, were used to determine plasma epinephrine and norepinephrine and serum insulin, glucose, FFA, glycerol, and FABP4 concentrations.

The plasma adrenaline and noradrenaline concentration were analyzed by high-performance liquid chromatography (Tosoh Corporation., Tokyo, Japan). The serum insulin concentration was measured via a chemiluminescent immunoassay (Abbott Japan, Tokyo, Japan). The serum TC and HDLC concentrations were measured via a direct enzymatic method (SEKISUI MEDICAL Corporation, Tokyo, Japan). The serum TG concentration was measured using the glycerol kinase–glycerol–3–phosphate oxidase erase method (Hitachi Chemical Diagnostics Systems Corporation., Tokyo, Japan). The serum Cre concentration was measured according to the endogenous creatine elimination reaction method (KANTO CHEMICAL Corporation, Tokyo, Japan). The estimated glomerular filtration rate (eGFR) was calculated using the equation for Japanese adult men (194 × creatinine concentration (mg/dl) ^−1.094^ × age (years) ^−0.287^). The serum glucose concentration was measured using an enzymatic method (Hitachi Chemical Diagnostics Systems Corporation., Tokyo, Japan). The serum TC concentration was measured using a cholesterol oxidase-peroxidase method (Hitachi Chemical Diagnostics Systems Corporation., Tokyo, Japan). The serum FFA concentration was measured using an enzymatic method (FUJIFILM Wako Pure Chemical Corp., Osaka, Japan). The serum glycerol concentration was analyzed via a coupled enzymatic reaction (Cayman Chemical, MI, USA). The serum FABP4 concentration was measured using an enzyme-linked immunosorbent assay kit (R&D Systems Inc., MN, USA). To eliminate inter-assay variation, samples from each participant were analyzed in the same run. Intra-assay CV of analysis for FFA, glycerol and FABP4 concentration was < 5.0%.

### Statistical analysis

The primary outcome was circulating FABP4 concentrations during the trials. Secondary outcomes included the effect of carbohydrate ingestion on circulating glycerol, FFA, insulin, and catecholamine concentrations. All data was obtained and there was no missing data. The Kolmogorov–Smirnov test and Levene’s test were used to confirm normality and homoscedasticity, respectively. In the case of non-normal distribution, a log transformation was performed. Glycerol, FFA, and FABP4 concentrations were statistically analyzed after log transformation. A paired-t test was used to determine differences in exercise intensity, EE, and substrate oxidation during exercise between the CE and FE trials. Two-way repeated-measures analysis of variance (trial × time) was used to compare changes in RER, and blood parameters between the two trials. When a significant interaction was observed, a Bonferroni post-hoc analysis was performed to determine differences between trials at a specific point in time. ES was calculated as Cohen’s *d* (small ≥ 0.20, medium ≥ 0.50, or large ≥ 0.80) for the post-hoc test. Pearson product-moment correlation coefficients were calculated to estimate the relationship between changes in glycerol and FFA levels during aerobic exercise (values after exercise– values before exercise, for ∆glycerol, and ∆FFA) and changes in circulating FABP4 concentrations following aerobic exercise (values after exercise– values 30 or 60 min after exercise, ∆30FABP4, and ∆60FABP4).

The sample size was calculated using the effect size (ES, *f* = 0.25) of the change in FABP4 concentration during exercise [18]. We determined that a sample size of at least nine would be required for approximately 80% power at 0.05 significance to detect an ES. The sample size was calculated using G*Power version 3.1.3 [29].

Statistical analyses were performed using SPSS version 28 software (IBM Corporation, Armonk, NY, USA). Data are presented as mean ± standard deviation (SD) or median (range). Values for blood parameters were adjusted according to plasma volume changes [27]. EE and carbohydrate and fat oxidation rates were calculated using VO_2_ and VCO_2_ [26]. Carbohydrate and fat oxidation rates were calculated using the following equations; Carbohydrate oxidation rate (g/min) = 4.585×VCO_2_ (L/min)– 3.226×VO_2_ (L/min), and Fat oxidation rate (g/min) = 1.695×VO_2_ (L/min)– 1.701×VCO_2_ (L/min). Statistical significance was set at *p* < 0.05.

## Results

### Participants characteristics

Thirteen young healthy men participated and completed this study (Table 1). None of the participants had regularly participated in sports or exercise within the previous six months. None of the participants had a history of metabolic, cardiovascular, or gastrointestinal disease. All participants were non-smokers and were not taking any medications or supplements known to affect lipid and carbohydrate metabolism. These details were confirmed through interviews during participant recruitment. During the trials, there were no adverse events.

**Table 1:** Characteristics of participants

Age (years)	22.2 (21–28)
Height (cm)	169.3 ± 5.7
Weight (kg)	66.7 ± 5.1
BMI (kg/m^2^)	23.3 ± 1.6
*Body composition*	
%Fat (%)	16.5 (12.6–28.8)
Fat mass (kg)	11.1 (8.2–20.5)
Skeletal muscle mass (kg)	52.0 ± 5.2
*Blood pressure*	
Systolic blood pressure (mmHg)	108.1 ± 8.7
Diastolic blood pressure (mmHg)	64.5 ± 7.7
*Health-related blood parameters*	
TC (mg/dL)	161.0 (132–244)
TG (mg/dL)	66 (40–139)
HDLC (mg/dL)	56.8 ± 11.2
LDLC (mg/dL)	94.6 ± 26.7
Creatinine (mg/dL)	0.89 ± 0.15
eGFR (mL/min/1.73m^2^)	87.1 ± 16.2
*Aerobic capacity*	
Maximal HR (beat/min)	187.4 ± 11.3
Maximal load (watts)	225 (165–240)
VO_2_peak (ml/kg/min)	38.1 ± 2.4
VO_2_peak (ml/min)	2564.2 ± 251.9

Values are presented as means ± SD or median (range). BMI, body mass index; %fat, percentage of fat; TC, total cholesterol; TG, triglycerides; HDLC, high-density lipoprotein cholesterol; LDLC, low-density lipoprotein cholesterol; eGFR, estimated glomerular filtration rate; HR, heart rate; VO_2_peak, peak oxygen uptake

### Exercise intensity and substrate oxidation

The changes in absolute and relative exercise intensity parameters, RER, EE, carbohydrate oxidation, and fat oxidation in each trial are shown in Table 2. The VO_2_ and %VO_2_ peak during exercise did not differ between the CE and FE trials (both *p* > 0.05). RER during the exercise was significantly higher in the CE trial than in the FE trial (*p* = 0.047, ES 0.61). EE during exercise did not differ between the CE and FE trials (CE 5.32 ± 0.46 kcal/min, FE 5.21 ± 0.46 kcal/min, *p* = 0.178, ES 0.39). Carbohydrate oxidation was significantly higher in the CE trial than in the FE trial (CE 0.93 ± 0.22 g/min, FE 0.77 ± 0.22 g/min, *p* = 0.011, ES 0.83), while fat oxidation was significantly lower in the CE trial than in the FE trial (CE 0.18 ± 0.09 g/min, FE 0.24 ± 0.10 g/min, *p* = 0.033, ES 0.67).

**Table 2:** Changes in expired gas and substrate oxidation during aerobic exercise in the CE, and FE trials

	Before Ex	10 min	20 min	30 min	40 min	p value			Mean ± SD (40-min exercise)
						Interaction	Trial	Time	
*Vo*_*2*_ *(mL/min)*									
CE	282 ± 60	1049 ± 129	1052 ± 116	1052 ± 107	1059 ± 87	0.679	0.352	**< 0.001**	1050 ± 96
FE	248 ± 58	1019 ± 136	1046 ± 127	1046 ± 80	1046 ± 80				1039 ± 91
*%Vo*_*2*_ *peak(%)*									
CE	11.1 ± 2.6	41.1 ± 5.3	41.3 ± 4.9	40.8 ± 4.8	41.5 ± 3.7	0.550	0.326	**< 0.001**	41.2 ± 4.2
FE	9.8 ± 2.6	39.9 ± 5.0	40.9 ± 4.1	41.1 ± 4.0	41.0 ± 3.3				40.7 ± 3.4
*RER*									
CE	0.94 ± 0.14*	0.92 ± 0.06	0.89 ± 0.06	0.88 ± 0.05	0.90 ± 0.04*	0.131	**0.014**	0.483	0.90 ± 0.05 *
FE	0.86 ± 0.14	0.89 ± 0.06	0.86 ± 0.05	0.87 ± 0.05	0.85 ± 0.06				0.87 ± 0.05
*Energy expenditure(kcal/min)*									
CE	1.58 ± 0.52	5.40 ± 0.67	5.34 ± 0.53	5.18 ± 0.51	5.37 ± 0.40	0.237	0.223	0.900	5.32 ± 0.46
FE	1.33 ± 0.33	5.20 ± 0.76	5.18 ± 0.62	5.25 ± 0.40	5.19 ± 0.40				5.21 ± 0.46
*Carbohydrate oxidation(g/min)*									
CE	0.28 ± 0.19	1.06 ± 0.31*	0.89 ± 0.27*	0.86 ± 0.22	0.95 ± 0.20 *	0.277	**0.011**	**0.014**	0.94 ± 0.22 *
FE	0.20 ± 0.16	0.84 ± 0.30	0.74 ± 0.24	0.77 ± 0.22	0.73 ± 0.26				0.77 ± 0.22
*Fat oxidation (g/min)*									
CE	0.05 ± 0.05	0.13 ± 0.11 *	0.20 ± 0.11	0.19 ± 0.07	0.18 ± 0.08 *	0.539	**0.003**	**0.029**	0.17 ± 0.08 *
FE	0.06 ± 0.04	0.21 ± 0.10	0.25 ± 0.10	0.24 ± 0.10	0.25 ± 0.11				0.24 ± 0.10

Values are presented as means ± standard deviation. Vo_2_, oxygen uptake; RER, respiratory exchange ratio; heart rate; CE, a carbohydrate ingestion exercise trial; FE, a fasted exercise trial. *, vs. FE (*p* < 0.05)

### Lipolytic-related hormones and metabolites

**Fig. 3:**
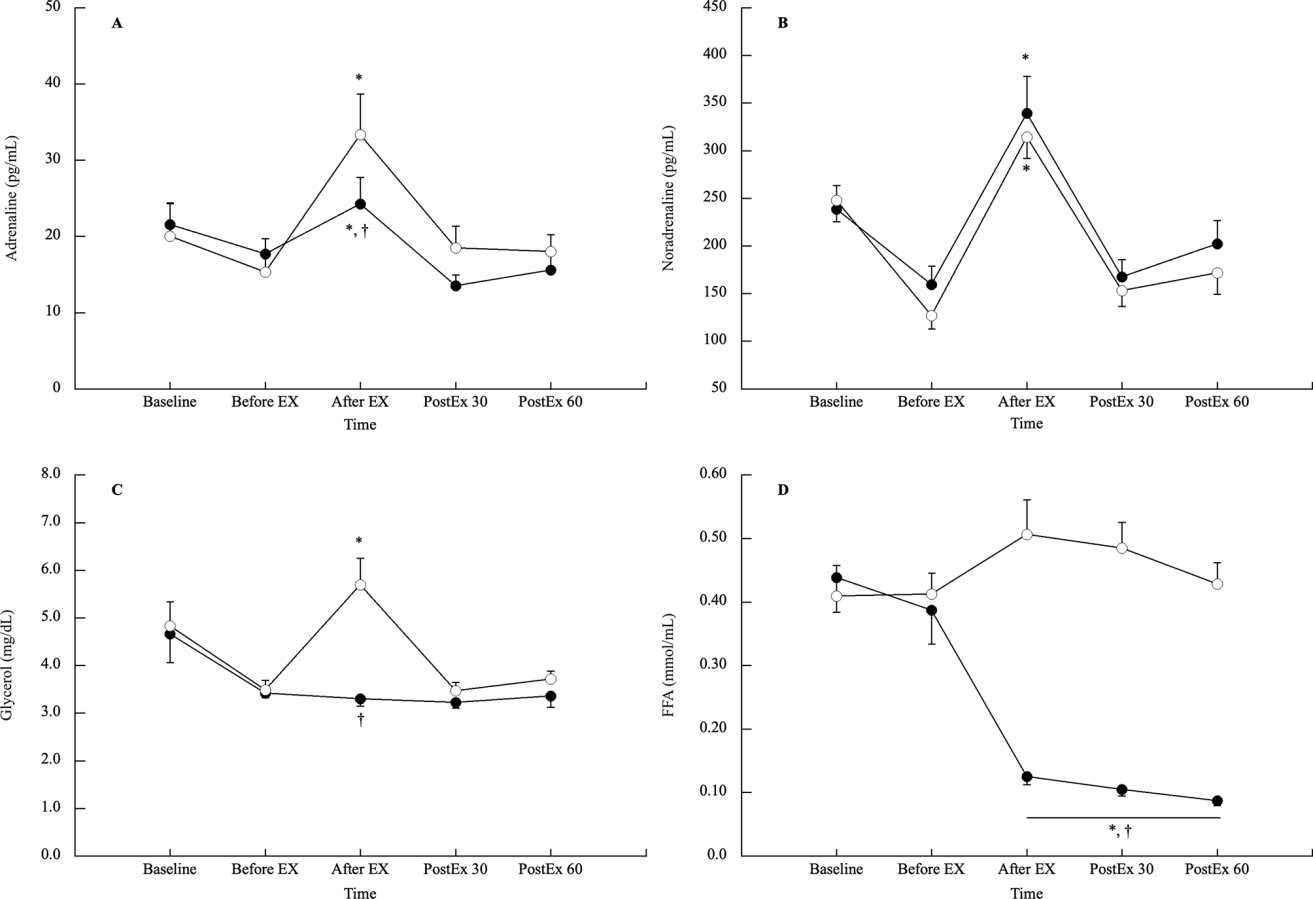
Lipolytic-related hormone and metabolite concentration response in the CE and FE trials (**A**: adrenaline, **B**: noradrenaline, **C**: glycerol, and **D**: FFA). Values are shown as mean ± SE. Solid circles and open circles are presented as the CE, and FE trials, respectively. * vs. before EX, † vs. FE trial at the same timepoint

Adrenaline, noradrenaline, FFA, and glycerol concentration responses are shown in Fig. 3. Adrenaline concentrations significantly increased immediately after exercise in both the CE and FE trials but were significantly higher in the FE trial than in the CE trials (*p* = 0.033, ES 0.66). Noradrenaline concentrations significantly increased immediately after exercise in both the CE and FE trials (*p* < 0.001). Post-exercise, both adrenaline, and noradrenaline returned to baseline values and did not differ between the two trials (*p* > 0.05). FFA concentrations significantly decreased immediately after exercise in the CE trial (*p* < 0.001, ES 2.63), but did not significantly change throughout the FE trial (*p* > 0.05). FFA concentrations remained significantly lower until 60 min post-exercise during the CE trial, at levels significantly lower than those of the FE trial (*p* < 0.001, ES > 3.20). The glycerol concentration did not change throughout the CE trial, whereas it increased significantly immediately after exercise in the FE trial (*p* = 0.005, ES 1.22). The glycerol concentration differed significantly between the two trials immediately after exercise (*p* < 0.001, ES 1.28).

### Glucose-related hormones and metabolites

**Fig. 4:**
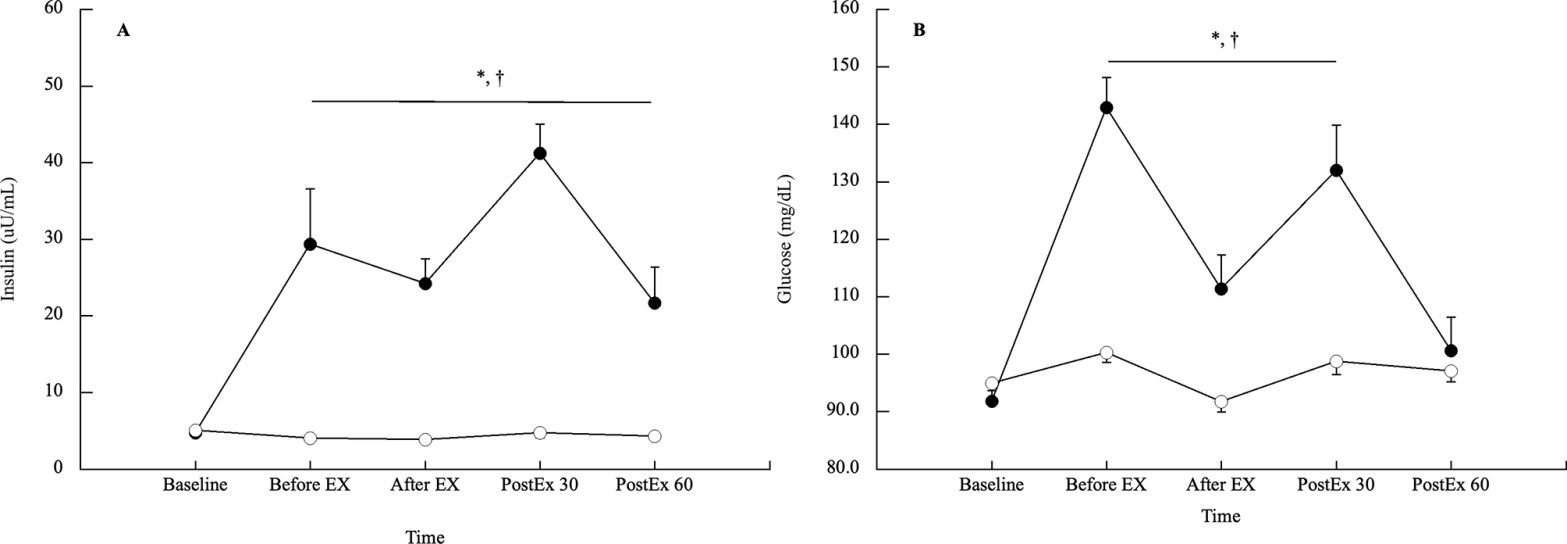
Glucose-related hormone and metabolite concentration response in the CE and FE trials (**A**: insulin, and **B**: glucose). Values are shown as mean ± SE. Solid circles and open circles are presented as the CE, and FE trials, respectively. * vs. before EX, † vs. FE trial at the same timepoint

Insulin and glucose concentration responses are shown in Fig. 4. Insulin concentrations increased significantly immediately before exercise in the CE trial (*p* < 0.001, ES 0.94), but did not change throughout the FE trial (*p* > 0.05). In addition, the insulin concentration remained significantly elevated until 60 min post-exercise in the CE trial, at levels significantly higher than those of the FE trial (*p* < 0.004, ES > 2.00). Similar to the insulin concentration, the glucose concentration significantly increased immediately before exercise in the CE trial (*p* < 0.001, ES 2.54). It remained significantly elevated until 30 min post-exercise in the CE trial, at levels significantly higher than those of the FE trial (*p* < 0.005, ES > 0.96).

### FABP4 concentration

**Fig. 5:**
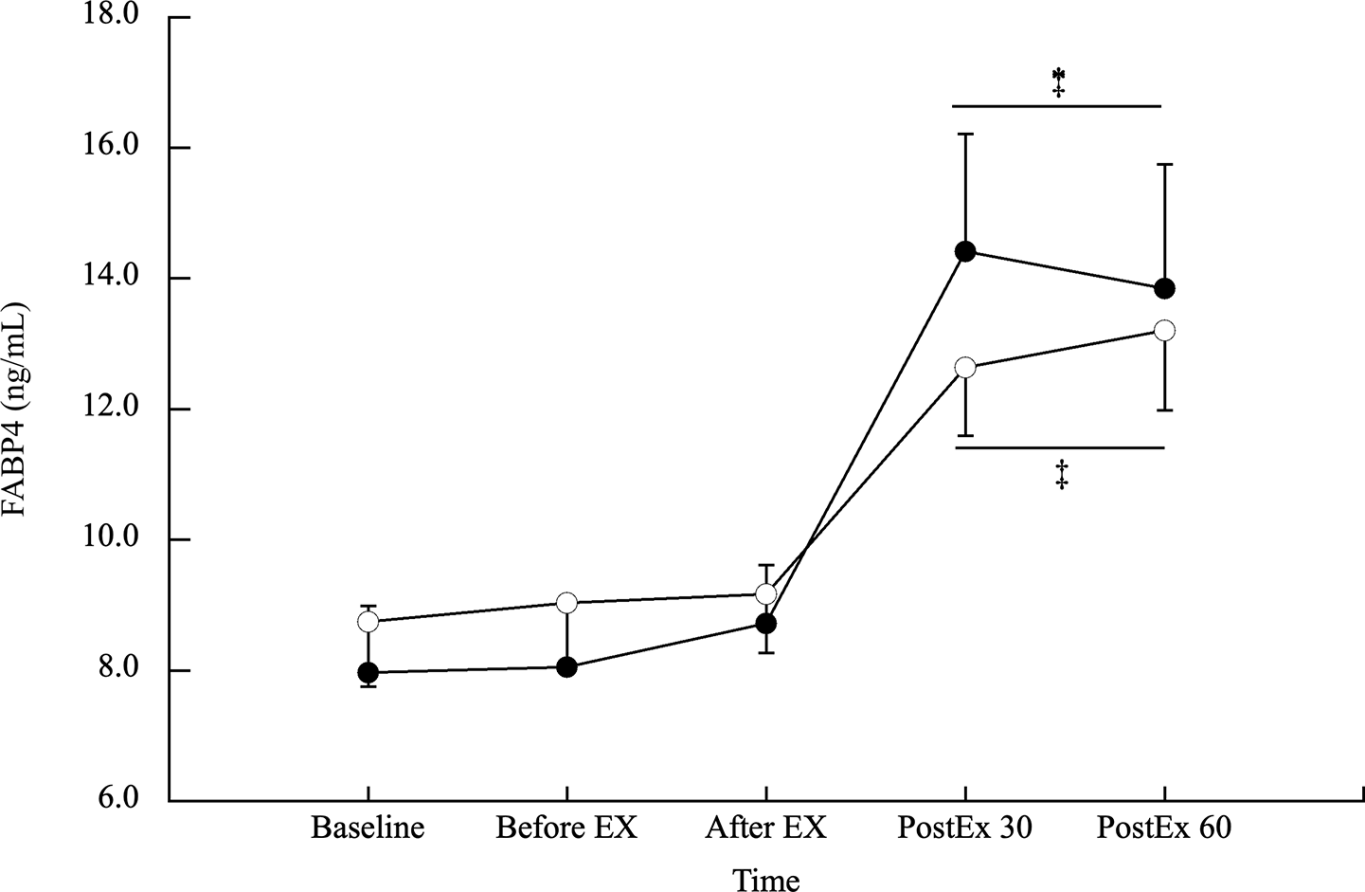
FABP4 concentration response in the CE and FE trials. Values are shown as mean ± SE. Solid circles and open circles are presented as the CE, and FE trials, respectively. ‡ vs. baseline, before EX, and after EX.

FABP4 concentration responses are shown in Fig. 5. FABP4 concentrations did not differ significantly between the CE and FE trials (*p* = 0.108). FABP4 concentration increased significantly by 77.9% and 62.0% 30- and 60-min post-exercise in the CE trial, respectively (*p* < 0.002, ES > 1.21). Similarly, it increased significantly by 64.3% and 63.2% 30- and 60-min post-exercise in the FE trial (*p* < 0.001, ES > 1.83), respectively. The magnitude of the increase in FABP4 concentration was similar between the CE and FE trials (*p* > 0.05).

### Correlation between changes in glycerol and FFA concentrations during aerobic exercise and changes in FABP4 concentration following aerobic exercise

Pearson’s moment correlation coefficients between changes in glycerol and FFA concentrations during aerobic exercise and changes in FABP4 concentration following aerobic exercise are shown in Table 3. There were no significant correlations between ∆glycerol, ∆FFA, and ∆30FABP4 (∆glycerol vs. ∆30FABP4: *r* = -0.044, *p* = 0.830, ∆FFA vs. ∆30FABP4: *r* = -0.197, *p* = 0.334). Further, no significant correlations were observed between ∆glycerol, ∆FFA, and ∆60FABP4 (∆glycerol vs. ∆60FABP4: *r* = 0.029, *p* = 0.887, ∆FFA vs. ∆60FABP4: *r* = -0.055, *p* = 0.790).

**Table 3:** Pearson’s moment product correlation coefficients between changes of glycerol, FFA concentrations during the acute aerobic exercise and change of FABP4 concentration post-exercise

∆BE	FABP4 (*n* = 26)		
	∆E30		∆E60
Glycerol	-0.044		0.029
P value	0.830		0.887
FFA	-0.197		-0.055
P value	0.334		0.790

∆BE, after exercise value– before exercise value; ∆E30, post EX30 min value-after exercise value; ∆E60, post EX60 min value-after exercise value

## Discussion

The main finding of the present study is that decreased glycerol and FFA and increased insulin concentration after carbohydrate ingestion do not suppress an increase in FABP4 concentrations post-aerobic exercise in healthy young men. Regardless of carbohydrate ingestion, the FABP4 concentration was unchanged during and after the aerobic exercise, but had increased from baseline by 30-min post-aerobic exercise. The extent of this increase was not influenced by carbohydrate ingestion. Further, changes in glycerol, and FFA concentrations during aerobic exercise did not correlate with changes in FABP4 concentration post-aerobic exercise. These findings suggest that suppression of lipolysis by elevation of insulin is not strongly involved in the increase in FABP4 secretion post-acute aerobic exercise.

Inhibition of adipose triglyceride lipase (ATGL) and hormone-sensitive lipase (HSL) activity is involved in the suppression of lipolysis [30, 31]. Since insulin inhibits ATGL and HSL activity [21], increased insulin concentrations after carbohydrate ingestion likely decreases ATGL and HSL activities. Decreased FABP4 is induced by the inhibition of ATGL and HSL activities [19]. Indeed, an increase in insulin secretion following ingestion of food decreases circulating FABP4 concentrations in humans [8, 32]. The results from previous studies suggest that decreased ATGL and HSL activity due to an elevation of insulin should suppress FABP4 secretion during and after aerobic exercise. Therefore, insulin elevation following carbohydrate ingestion should decrease circulating FABP4 concentrations during and after aerobic exercise. However, despite higher insulin and lower adrenaline concentrations during aerobic exercise in the CE trial, the FABP4 concentration was not suppressed and still increased after aerobic exercise in the CE trial. This FABP4 response was almost the same as that observed in the FE trial. Additionally, changes in glycerol and FFA concentrations during aerobic exercise did not correlate with FABP4 concentrations after aerobic exercise. The results suggest that suppression of lipolysis by insulin elevation following carbohydrate ingestion does not contribute to a high degree to increased circulating FABP4 concentrations following aerobic exercise.

Although the reasons for the lack of effect of carbohydrate ingestion on the change in FABP4 concentration remain unclear, an increase in adrenaline and noradrenaline during aerobic exercise may have had an effect. Elevation of insulin concentrations is followed by a decrease in circulating FABP4 concentrations following meal ingestion in the resting state [8, 32]. In the present study, elevated insulin concentrations were comparable to those seen in previous studies [8, 32]. However, the secretion of adrenalin and noradrenaline, as well as that of insulin, increases during aerobic exercise following carbohydrate ingestion [33]. Therefore, the stimulation of lipolysis by exercise-induced adrenalin and noradrenaline secretion counteracts at least partly the suppression of lipolysis by insulin compared to in a non-exercise resting state [34]. Although ATGL and HSL activation during aerobic exercise is assumed to be suppressed by increases in insulin [21], the suppression of ATGL and HSL activation during exercise may be weaker than that in a non-exercise resting state. In the CE trial, there was a sufficient increase in insulin during exercise, however, ATGL and HSL activation may not be impaired sufficiently to affect differences in FABP4 secretion from adipocytes.

Increased circulating FABP4 concentrations have been observed following aerobic exercise [16, 18], and our findings support the results of those studies [16, 18]. However, the underlying mechanisms remain unclear. Several pieces of evidence have demonstrated that FABP4 secretion from adipocytes into the circulation is regulated by signaling pathways relating to the activation of lipolysis, which is mediated by β-adrenergic receptor-mediated adenyl cyclase-protein kinase A and natriuretic peptide receptor-A-mediated guanylyl cyclase-protein kinase G pathways [7, 8, 19, 20]. Moreover, the secretion of adrenaline, noradrenaline, and adrenal natriuretic peptides decreases rapidly after aerobic exercise [35–37]. It is thus unlikely that the increased circulating FABP4 concentration following aerobic exercise is due to the activation of a lipolytic signal following aerobic exercise. Considering the mechanism of FABP4 secretion and the result of the present study, it is reasonable to assume that activation of the lipolytic signal, rather than lipolysis per se, during aerobic exercise leads to an increase in the circulating FABP4 concentration after aerobic exercise. Additionally, despite the high insulin concentration after acute exercise in the CE trial, circulating FABP4 concentrations were not suppressed. Hence, we speculate that there is a time delay for FABP4 secretion from adipocytes to be reflected in blood levels. In a previous study [8], even after postprandial insulin levels peaked and declined, FABP4 concentration did not increase and remained at a low level. Thus, we believe that FABP4 secretion increased gradually with increased lipolytic signal activation after the start of aerobic exercise, and this was reflected in circulating FABP4 concentrations following aerobic exercise.

In contrast, circulating FABP4 concentrations did not change during aerobic exercise in both the CE and FE trials. The result is not in agreement with the previous studies [16, 17]. The discrepancy may be accounted for by the difference in exercise intensity performed. In fact, previous studies have observed that FABP4 concentration does not increase during acute exercise below anaerobic threshold [16] and low-intensity aerobic exercise [18] while it increases during acute aerobic exercise above anaerobic threshold [16] and incremental maximal exercise [17]. Adrenaline, noradrenaline, and atrial natriuretic peptides, which stimulate lipolytic pathway, increase during aerobic exercise depending on exercise intensity [36]. Since FABP4 secretion is regulated by the activation of lipolytic pathway [7, 8, 19, 20], it is possible that aerobic exercise corresponding relatively higher intensity lead to promoting FABP4 secretion during aerobic exercise.

Increased insulin secretion induced by ingestion of a meal before aerobic exercise suppresses lipolysis during aerobic exercise [22, 23]. However, if meals are not ingested multiple times before or during aerobic exercise, there is a possibility that the inhibitory effect of insulin on lipolysis may not be sustained during the exercise [37]. As the present study aimed to examine the influence of insulin suppression of lipolysis on FABP4 concentrations during and after exercise, we needed a study design that could suppress lipolysis more effectively. Therefore, we arranged for participants to ingest carbohydrates not only 30 min before the start of exercise but also immediately before and 20 min after the start of exercise. Additionally, aerobic exercise intensity was set at low (40%Vo_2_peak) because an increased circulating FABP4 concentration is expected not during but after low- to moderate intensity aerobic exercise [18], and insulin release is prevented during moderate- to vigorous aerobic exercise [23]. The insulin concentration remained high during and after aerobic exercise in the CE trial. When insulin concentrations remain high, lipolysis does not increase during low-intensity aerobic exercise [23]. Moreover, even when carbohydrates are ingested 60 min before exercise, the rate of lipolysis during aerobic exercise is inhibited by nearly 50% compared to in the fasting state [22]. It is likely that lipolysis was suppressed during aerobic exercise in the CE trial, as seen in previous studies [22, 23]. In the CE trial, glycerol concentrations did not increase, whereas FFA concentrations decreased. Although these parameters do not completely account for lipolysis, the rate of re-esterification is very low during aerobic exercise [38], and re-esterification within adipocytes is unaffected by an increase in insulin concentrations [39]. Glycerol and FFA concentration responses are thus expected to reflect the suppression of lipolysis.

The present study has several limitations. First, the sample size was relatively small in the present study (*n* = 13). Nevertheless, there was a high ES (Cohen’s *d* > 1.21) with adequate statistical power (> 99%) for the significant increase in FABP4 concentration after acute exercise observed in this study. Second, the participants were young healthy men; therefore, our findings may not apply to disease patients, women, adolescents, and older adults. FABP4 concentrations are associated with the amount of body fat [40–42] and lipolytic response [7, 8, 19, 20]. Given the variations in sex, age, and pathological conditions influencing body fat accumulation and hormonal fluctuations (by menstrual cycle, growth, and aging), it cannot be denied that the change in FABP4 concentration in response to exercise may differ. Therefore, our findings may not directly generalize to disease patients, women, adolescents, and older adults. Third, we did not include no-exercise controls, such as carbohydrate ingestion + no exercise or fasting + no exercise trials. However, previous studies have shown that circulating FABP4 concentrations decrease at least 1 h after food ingestion [8]. We have also confirmed that FABP4 does not change during fasting during the same period as in the present study [18]. Fourth, the experimental protocol in the present study had a duration of only 100 min. Further investigations with longer post-exercise periods may be required to follow longer-scale changes in circulating FABP4 concentrations.

In conclusion, the present study demonstrated that carbohydrate ingestion did not suppress circulating FABP4 levels during and after aerobic exercise in healthy men. Further, no correlation was observed between the magnitude of changes in FABP4, glycerol, and FFA concentrations during and after aerobic exercise. These findings indicate that suppression of lipolysis by insulin elevation after carbohydrate ingestion does not contribute strongly to increased circulating FABP4 concentration following aerobic exercise.

## Data Availability

All data generated or analyzed during this study are included in this published article.

## Abbreviations

FABP4: fatty acid binding protein 4
CE trial: a carbohydrate ingestion exercise trial
FE trial: a fasted exercise trial
VO_2_peak: peak oxygen uptake
VO_2_: oxygen uptake
VCO_2_: carbon dioxide output
RER: respiratory exchange ratio
EDTA: ethylenediaminetetraacetic acid
TC: total cholesterol
HDLC: high-density lipoprotein cholesterol
TG: triglyceride
Cre: creatinine
FFA: free fatty acid
CV: coefficient of variation
ES: effect size
SD: standard deviation
